# The Reproducibility and Reliability of Insulin Sensitivity and Secretion Indices in Children and Adolescents

**DOI:** 10.1155/2024/2136173

**Published:** 2024-04-30

**Authors:** Nellie Said Hani, Mary Ellen Vajravelu, Jennifer L Meijer, Harlan McCaffery, Julie Sturza, Emily Dhadphale, Joyce M Lee

**Affiliations:** ^1^Department of Pediatrics, Division of Pediatric Endocrinology, University of Michigan, Ann Arbor, USA; ^2^Division of Pediatric Endocrinology, Diabetes and Metabolism, UPMC—Children's Hospital of Pittsburgh, Pittsburgh, USA; ^3^Geisel School of Medicine, Department of Pediatrics, Dartmouth College, Hanover, USA; ^4^Susan B. Meister Child Health Evaluation and Research Center, Division of Pediatric Endocrinology, University of Michigan, Ann Arbor, USA

## Abstract

**Objective:**

To determine whether fasting or oral glucose tolerance test (OGTT)-derived insulin measures would be more reproducible and whether there would be differences based on weight, sex, race, and pubertal status.

**Design:**

Observational study. *Setting*. Clinical research unit. *Patients or Other Participants*. Two hundred fifty-seven overweight/obese (BMI ≥ 85th%, *n* = 186) and normal weight (BMI < 85th%, *n* = 71) children without diabetes between ages of 8 and 17 were included in the study.

**Methods:**

OGTT tests performed in study participants at two separate visits within a 3-week period. We performed two formal oral glucose tolerance tests within a 3-week period. The reproducibility of fasting measures was compared with OGTT-derived measures by weight categories and compared by weight, sex, race, and pubertal status. Comparisons were made between the correlation coefficients of fasting vs. OGTT-derived measures and between normal weight vs. obese/overweight participants, male vs. female, White vs. Black, and pre- vs. post-midpubertal. Intraclass correlation coefficients were calculated for each comparison as well.

**Results:**

For insulin sensitivity, the OGTT-derived measure was more reproducible than the fasting measures. There were no significant differences in reproducibility in the overweight/obese population compared to the normal weight population nor by sex, race, or pubertal status.

**Conclusions:**

Nonfasting insulin sensitivity measures are more reproducible than fasting insulin sensitivity measures, regardless of weight category. Insulin secretion measures have poor reproducibility overall. Weight status, sex, race, and midpubertal stage do not impact the reproducibility of insulin sensitivity and secretion measures.

## 1. Introduction

With the increasing prevalence of childhood obesity, there has been a rise in obesity-related prediabetes and type 2 diabetes (T2D). The incidence of pediatric T2D has increased by 7.1% annually between 2001 and 2012, and the prevalence of prediabetes has increased from 11.6% in 1999–2002 to 28.2% in 2015–2018 [1, 2]. The early stages of developing prediabetes are defined by alterations in pancreatic beta-cell secretion of insulin and reduced insulin sensitivity at the cellular level [3]. Reduced insulin sensitivity has been shown to confer a risk to developing prediabetes in the pediatric population [4]. Given the increased prevalence of pediatric prediabetes and the more aggressive natural history of T2D in pediatrics compared with adults, it is necessary to identify children at risk of developing prediabetes prior to disease onset [4]. Thus, it is important to identify effective and precise screening tools for prediabetes in the pediatric population.

The gold standard tests for insulin sensitivity and secretion evaluation are the hyperinsulinemic–euglycemic clamp (HEC) and hyperglycemic clamp, respectively [5]. These are valuable research tools but are clinically impractical because they are too burdensome and invasive for standard measurement. As a result, insulin sensitivity and insulin secretion indices derived from fasting measures or oral glucose tolerance tests have been developed and validated in pediatrics as surrogate markers [6, 7]. OGTT-derived indices represent the efficiency of total body glucose utilization, taking into account both hepatic and muscle insulin sensitivities [8]. In contrast, indices that only use fasting measures are more reflective of hepatic insulin sensitivity [9]. Insulin sensitivity indices include the Matsuda index, the Quantitative Insulin Sensitivity Check Index (QUICKI), and the Homeostatic Model Assessment for Insulin Resistance (HOMA-IR) [10–12]. Insulin secretion indices include the insulinogenic index, disposition index, and Homeostasis Model Assessment of Beta Cell Function (HOMA-beta) [12–14].

There is a paucity of data investigating test–retest reliability of the aforementioned surrogate measures of insulin sensitivity and secretion in children and adolescents. More specifically, the reproducibility of these measures at different stages of puberty, a time of increased insulin resistance, and across racial and ethnic groups and by sex require additional study, as overweight and obesity affects diverse pediatric populations [15–18]. To our knowledge, the few studies which have evaluated reproducibility of insulin sensitivity and secretion have been conducted in a non-US population, in adults, or in pediatric populations with few participants [19–21].

The objective of our study was to assess the reproducibility of a variety of indices of insulin sensitivity and secretion in adolescents using repeat measures within 1 month, assessing overall reproducibility and differences by race, sex, weight status, and pubertal status. We defined reproducibility as the closeness of agreement between results of the same measurement when obtained at two different time points.

## 2. Methods

The study population consisted of a sample of youth 8–17 years old with normal weight (body mass index (BMI) percentile  < 85th) or overweight/obesity (BMI percentile ≥ 85th) who were recruited from primary care pediatric specialty clinics in southeast Michigan. We excluded individuals who had known diabetes, which was determined from medical chart review and/or self-report at the time of study screening and enrollment. Patients who were using medications known to affect glucose metabolism or who were pregnant were also excluded. We did not screen for prediabetes or dysglycemia prior to enrollment in the study. We reevaluated study participants for both dysglycemia and prediabetes based on their OGTT results at both visits.

Participants attended two study visits at the Michigan Clinical Research Unit, where a medical history, vital signs, anthropometry, and laboratory evaluation were performed. The two visits occurred at a median of 14 days (interquartile range 7–28) apart. At each of the visits, participants were requested to fast for a minimum of 12 hr prior to a 2-hr oral glucose tolerance test (OGTT), in which venous blood samples were drawn at baseline and every 30 min up to 2 hr after glucose load (1.75 g/kg up to maximum of 75 g). Participants were instructed to consume the glucose solution within 5 min, and they were not instructed to do any carbohydrate loading.

### 2.1. Study Definitions

In our study, dysglycemia was defined as having prediabetes (fasting plasma glucose 100–125 mg/dL or 2-hr plasma glucose 140–199 mg/dL) or diabetes (fasting plasma glucose 126 mg/dL or higher or 2-hr plasma glucose 200 mg/dL or higher) via an OGTT at either visit 1 or 2 [22].

We calculated measures of insulin sensitivity and secretion, with formulas listed below. *G* and *I* refer to the glucose and insulin measurements obtained at the specified time point during the OGTT, indicated by the subscripted number.

(a) Insulin sensitivity measures:
  - Homeostasis model assessment of insulin resistance (HOMA-IR): $\frac{G_{0}\times I_{0}}{22.5}$ [12].
  - Whole body sensitivity index (WBISI): $\frac{10000}{\sqrt{\left(G_{0}\times I_{0}\right)\times \left(\frac{G_{0}+G_{120}}{2}\times \frac{I_{0}+I_{120}}{2}\right)}}$ [10].
  - Quantitative insulin sensitivity check index (QUICKI): $\frac{1}{\log I_{0}+\log G_{0}}$ [11].
  - 1/fasting insulin levels.
  - Fasting glucose to insulin ratio (FGIR).
(b) Insulin secretion measures:
  - Insulinogenic index (IGI): $\frac{\Delta I_{0,30}}{\Delta G_{0,30}}$ [13].
  - Homeostasis model assessment of beta cell function (HOMA-B): $\frac{20\times I_{0}}{G_{0}-3.5}$ [12].
  - Disposition index (DI): $\frac{\Delta I_{0,30}}{\Delta G_{0,30}}\times \frac{1}{I_{0}}$ [23].

Measured height and weight were converted to BMI percentiles for age and sex according to the 2000 United States Centers for Disease Control and Prevention growth curves.

Covariates of interest included sex; race; classified as Black, White, or other; and pubertal status. Pubertal status was classified as either pre-midpuberty (<14 years old in boys and <12 years old in girls) based on the average age at peak height velocity curves created by Tanner et al. [24] or post-midpuberty (≥14 years old in boys and ≥12 years old in girls) [25]. Tanner staging was not available for all subjects and thus was not used to classify puberty.

### 2.2. Outcomes and Analysis

Our outcomes of interest were the reproducibility (Spearman correlation coefficient and intraclass correlation coefficient (ICC)) of individual measures of insulin sensitivity (HOMA-IR, WBISI, QUICKI, 1/fasting insulin, FGIR) and secretion (IGI, DI, HOMA-B). Given that the data fit a non-normal distribution, median values rather than means at each visit were computed for each test at visits 1 and 2.

Univariate statistics demonstrated the skewed distribution of the test values. Spearman correlations and ICCs of the first and second measurements were calculated for each test. We followed the method used by similar studies to determine the ICCs, namely, running at repeated measures ANOVA and calculating ICC as the ratio of the variability in the measure between participants over the total variation in the measure from all sources [21, 26, 27]. Higher values for the ICC indicate higher reproducibility of the measure. The 95% confidence intervals for the ICC values were calculated using a publicly available SAS macro [28]. We also examined comparisons of reproducibility of fasting vs. OGTT-derived measures by weight category via correlation coefficient comparisons, which were performed using the method of Dunn and Clark [29]. Finally, we examined comparisons of reproducibility of each insulin secretion and sensitivity measure between overweight/obese vs. normal weight groups, mid-pubertal vs. post-pubertal, male vs. female, and Black vs. white subjects using Fisher's *z*-transformation to compare the correlation coefficients between the patient groups. Due to few participants of race other than Black or White, we limited race comparisons to these two groups of children. Analysis was done using R version 4.0.5 and the package *cocor* and SAS 9.4. Since we conducted multiple comparisons, we used a Bonferroni correction; results were considered significant for *p* value of <0.001 (original alpha of 0.05 divided by the number of comparisons, 50).

Laboratory analyses were performed by the Michigan Diabetes Research Center (MDRC) Core Laboratories. Glucose was measured using the glucose hexokinase method. Glucose assays were run on a Randox rX Daytona chemistry analyzer (Randox Laboratories Limited, United Kingdom). Insulin was profiled using a double-antibody radioimmunoassay using an 125I-Human insulin tracer (Linco Research), a guinea pig anti-porcine insulin first antibody (MDRTC, 68.5% cross-reaction to human proinsulin), and a goat anti-guinea pig gamma globulin (Antibodies Inc.)–PEG second antibody and standardized against the Human Insulin International Reference Preparation (NIBSC). The limit of sensitivity for the assay is 2.1 *µ*U/ml. Interassay and intraassay variabilities are 3.8% and 2.7%, respectively, at 25 *µ*U/ml. The interassay coefficients of variation were less than 10% across multiple levels, spanning from approximately 7–150 *µ*U/ml [30]. HbA1c was determined using a Tosoh G7 HPLC Analyzer (Tosoh Biosciences Inc, South San Francisco, CA).

This study was approved by the University of Michigan Institutional Review Board. Written informed consent was obtained from the parent/guardian for all participants, and participants 10 years or older provided written assent.

## 3. Results

### 3.1. Subjects

A total of 257 adolescents between the ages of 8 and 17 years were included in the study, of whom 186 were overweight/obese and 71 were normal weight. Table S1 includes a breakdown of our patient population by sex, race, and pubertal stage. Overall, 8% (*n* = 21) of subjects demonstrated dysglycemia (meeting prediabetes or diabetes criteria) during their OGTT either at visit 1 or visit 2. Twenty-four percent (*n* = 5) of these subjects were of normal weight, and 76% (*n* = 16) were overweight/obese.

Table 1 shows the median values and the interquartile range for each insulin sensitivity and secretion measure, as well as for glucose and insulin at time 0 and 120 at both visits.  Table 2 shows the ICCs and comparison of Spearman correlation coefficients for fasting and OGTT-derived sensitivity/secretion indices for the overall population and stratified by normal weight vs. overweight/obesity status. Based on the comparison of Spearman correlations, the OGTT-derived sensitivity measurement (WBISI) had greater reproducibility compared with HOMA-IR, QUICKI, FGIR, and 1/fasting insulin for the overall, overweight/obese groups. WBISI also had greater reproducibility than HOMA-IR and QUICKI in the normal weight group, but not when compared to FGIR or 1/fasting insulin. There were no significant differences between HOMA-B and IGI or DI for all groups.

Table 3 shows the Spearman correlations and ICCs for each measure and the comparisons of the Spearman correlations between each categorical variable subset: OV/OB vs. normal weight, Black vs. White, male vs. female, and pre-midpuberty and post-midpuberty, with *p* values representing the significance of the difference between the Spearman correlations. There were no significant differences in the reproducibility of measures for all comparison groups.

## 4. Discussion

To our knowledge, this is the largest study to date to evaluate the reproducibility of insulin sensitivity and insulin secretion measures in adolescents and to compare reproducibility by weight status, race, sex, and mid-pubertal stage. Given the known variability of postchallenge glucose measures, we had expected less variability with fasting measures compared to OGTT-derived measures, both for sensitivity and secretion [21]. Therefore, our finding that postchallenge insulin measures of sensitivity were generally more reproducible than fasting insulin sensitivity measures among overall, overweight/obese, and normal weight groups was surprising. We are unsure of why this is the case. We had also anticipated differences in the reproducibility of insulin measures, particularly insulin sensitivity measures, between sex, race, and pubertal categories, but these hypotheses were not borne out with our analysis [15–18].

We are aware of two studies that have examined the reproducibility of insulin sensitivity and secretion measures in the pediatric population [20, 21]. Cockcroft et al. [20] examined the reliability—expressed through coefficients of variation—of fasting measures in a group of 28 preadolescent and adolescent boys and of OGTT-derived measures in a subset of eight boys who had an average BMI of 21.8. Notable results included a lower coefficient of variation for QUICKI and FGIR for fasting measures, but the authors did not include comparisons of the reproducibility of each of the measures. Furthermore, the study was limited by its small sample size, was based on a homogeneous study population of only males in England, and did not account for variations in BMI weight status. Libman et al. [21] focused on the reproducibility of the OGTT, fasting plasma glucose, and 2-hr glucose in 60 overweight youth and not on insulin sensitivity measures.

A recent adult study by Hudak et al. [19] looked at the reproducibility of insulin sensitivity and insulin secretion indices in adults. The authors recruited a cohort of 89 adults without diabetes who underwent two repeat OGTT's and evaluated the relative reproducibility of fasting measures vs. postchallenge OGTT-derived measures, by comparing coefficients of variation. They found that for insulin sensitivity fasting indices, the revised QUICKI and original QUICKI showed lower coefficients of variation than other fasting indices, suggesting more reproducibility, and they found that indices based on fasting variables had smaller coefficients of variation than those derived from OGTTs. We are not able to directly compare our results to those of Hudak et al. [19] due to different statistical methods, but we did find that overall, our OGTT-derived insulin sensitivity measures were more reproducible than fasting measures, suggesting that there may be differences in reproducibility for pediatric vs. adult populations.

Innovations of our study include the relatively large sample size, diversity in age and race, the inclusion of overweight and obese but also a substantial population of children who are normal weight, and the inclusion of fasting vs. OGTT-derived measures. However, we do acknowledge limitations of our study, which includes the fact that the majority of children were pubertal at the time of testing. We did account for the mid-pubertal growth spurt by using age as a proxy, but there may be differences in reproducibility for these measures pre- and postpubertal which we could not account for. We did not perform a physical exam for Tanner staging and therefore used age instead, but we acknowledge that pubertal staging by age may not be as reliable in overweight/obese children, as there is a known influence of adiposity on earlier pubertal development [31]. The children in the study did not have diabetes based on the OGTT, but we acknowledge there could be differences in reproducibility for children who are on the verge of developing type 2 diabetes. The rates of prediabetes defined by OGTT in the population were very small, so we could not perform a comparison based on prediabetes status. Because of the low rates of prediabetes, we did not separate out the overweight and obese groups with prediabetes. Finally, we did not perform gold standard clamp studies to evaluate insulin sensitivity and secretion, but this would be impractical in a population of this size.

## 5. Conclusion

Additional studies in adolescents are needed to assess the reproducibility of insulin sensitivity and secretion indices, as these indices are important for informing clinical and epidemiological research studies evaluating future diabetes risk.

## Figures and Tables

**Table 1:** Medians and interquartile ranges for each insulin secretion, insulin sensitivity, and OGTT measures.

Category	Measures	Visit 1—median (IQR)	Visit 2—median (IQR)
Insulin sensitivity	HOMA-IR	3.67 (2.43–5.28)	3.29 (2.28–4.85)
	QUICKI	0.14 (0.13–0.15)	0.14 (0.13–0.15)
	FGIR	4.94 (3.54–7.18)	5.59 (3.56–7.76)
	1/fasting insulin	0.06 (0.04–0.09)	0.06 (0.04–0.09)
	WBISI	2.84 (1.90–4.22)	2.97 (1.98–4.66)

Insulin secretion	HOMA-B	284.87 (189.00–416.57)	259.71 (185.14–406.29)
	IGI	2.82 (1.55–4.66)	2.82 (1.59–4.72)
	DI	7.18 (4.77–11.43)	7.58 (5.31–11.86)

OGTT measures	G0	86 (81–91)	86 (80–90)
	G120	101 (85–113)	97 (85–112)
	I0	17.2 (11.6–23.2)	15.9 (11.0–23.0)
	I120	73.4 (45.0–119.5)	67.1 (42.0–114.9)

For each measure of insulin sensitivity, insulin secretion, and OGTT-derived glucose and insulin at time 0 min and 120 min, we state the median value with the interquartile range (IQR) at each visit.

**Table 2:** Spearman correlations and ICCs of each fasting and OGTT-derived insulin sensitivity measure between visits 1 and 2 and the *p* value of the Spearman correlation comparison between fasting vs. OGTT-derived measures.

Measurement comparison			All participants					Overweight/obese					Normal weight				
	Fasting measure	OGTT-derived measure	Fasting correlation (V1 vs. V2)	OGTT-derived correlation (V1 vs. V2)	*p* Value	Fasting ICC (V1 vs. V2)	OGTT-derived ICC (V1 vs. V2)	Fasting correlation (V1 vs. V2)	OGTT-derived correlation (V1 vs. V2)	*p* Value	Fasting ICC (V1 vs. V2)	OGTT-derived ICC (V1 vs. V2)	Fasting correlation (V1 vs. V2)	OGTT-derived correlation (V1 vs. V2)	*p* Value	Fasting ICC (V1 vs. V2)	OGTT-derived ICC (V1 vs. V2)
Insulin sensitivity	HOMA-IR	WBISI	0.73 (0.66–0.78)	0.83 (0.79–0.87)	<0.001 ^*∗*^	0.73 (0.67–0.78)	0.80 (0.75–0.84)	0.72 (0.64–0.78)	0.80 (0.74–0.84)	<0.001 ^*∗*^	0.71 (0.63–0.77)	0.75 (0.68–0.81)	0.53 (0.34–0.68)	0.83 (0.74–0.89)	<0.001 ^*∗*^	0.50 (0.31–0.65)	0.79 (0.68–0.86)
	QUICKI	WBISI	0.73 (0.66–0.78)	0.83 (0.79–0.87)	<0.001 ^*∗*^	0.72 (0.66–0.77)	0.80 (0.75–0.84)	0.72 (0.64–0.78)	0.80 (0.74–0.84)	<0.001 ^*∗*^	0.73 (0.66–0.79)	0.75 (0.68–0.81	0.53 (0.34–0.68)	0.83 (0.74–0.89)	<0.001 ^*∗*^	0.50 (0.31–0.65)	0.79 (0.68–0.86)
	FGIR	WBISI	0.76 (0.71–0.81)	0.83 (0.79–0.87)	<0.001 ^*∗*^	0.67 (0.60–0.73)	0.80 (0.75–0.84)	0.75 (0.68–0.81)	0.80 (0.74–0.84)	<0.001 ^*∗*^	0.70 (0.62–0.77)	0.75 (0.68–0.81	0.58 (0.40–0.71)	0.83 (0.74–0.89)	0.08	0.48 (0.28–0.64)	0.79 (0.68–0.86)
	1/fasting insulin	WBISI	0.75 (0.70–0.80)	0.83 (0.79–0.87)	<0.001 ^*∗*^	0.66 (0.58–0.72)	0.80 (0.75–0.84)	0.75 (0.68–0.81)	0.80 (0.74–0.84)	<0.001 ^*∗*^	0.69 (0.61–0.76)	0.75 (0.68–0.81	0.54 (0.35–0.69)	0.83 (0.74–0.89)	0.03	0.46 (0.26–0.62)	0.79 (0.68–0.86)

Insulin secretion	HOMA-B	IGI	0.69 (0.62–0.75)	0.61 (0.53–0.68)	0.08	0.30 (0.18–0.41)	−0.01 (−0.13–0.11)	0.68 (0.59–0.75)	0.56 (0.45–0.65)	0.72	0.26 (0.12–0.39)	−0.04 (−0.18–0.10)	0.61 (0.44–0.74)	0.65 (0.49–0.77)	0.05	0.60 (0.43–0.73)	0.31 (0.09–0.50)
	HOMA-B	DI	0.69 (0.62–0.75)	0.52 (0.42–0.60)	0.002	0.30 (0.18–0.41)	−0.06 (−0.18–0.06)	0.68 (0.59–0.75)	0.49 (0.37–0.59)	0.77	0.26 (0.12–0.39)	−0.11 (−0.25–0.03)	0.61 (0.44–0.74)	0.58 (0.40–0.72)	0.005	0.60 (0.43–0.73)	0.13 (−0.10–0.35)

Comparisons of Spearman correlations were performed overall and then specifically for overweight/obese participants and normal weight participants.  ^*∗*^*p* <  0.001.

**Table 3:** Spearman correlations and ICCs of insulin sensitivity and secretion measures between visits 1 and 2, and *p* values of the Spearman correlation comparison between overweight/obese vs. normal weight, Black vs. White, male vs. female, pre-midpuberty vs. post-midpubertal children.

Category	Measures	Overweight/obese vs. normal weight					Black vs. White					Male vs. female					Pre- vs. post-midpuberty				
		OV/OB correlation	Normal weight correlation	*p*-Value	OV/OB ICC	Normal weight ICC	Black correlation	White correlation	*p*-Value	Black ICC	White ICC	Male correlation	Female correlation	*p*-Value	Male ICC	Female ICC	Pre-midpuberty correlation	Post-midpuberty correlation	*p*-Value	Pre-midpuberty ICC	Post-midpuberty ICC
Insulin sensitivity	HOMA-IR	0.72 (0.64–0.78)	0.53 (0.34–0.68)	0.03	0.71 (0.63–0.77)	0.50 (0.31–0.65)	0.76 (0.63–0.85)	0.74 (0.66–0.81)	0.83	0.38 (0.15–0.57)	0.82 (0.76–0.87)	0.72 (0.61–0.79)	0.72 (0.63–0.79)	0.91	0.83 (0.76–0.88)	0.66 (0.56–0.74)	0.73 (0.61–0.82)	0.73 (0.65–0.79)	0.97	0.63 (0.48–0.75)	0.79 (0.73–0.84)
	QUICKI	0.72 (0.64–0.78)	0.53 (0.34–0.68)	0.03	0.73 (0.66–0.79)	0.50 (0.31–0.65)	0.76 (0.63–0.85)	0.74 (0.66–0.81)	0.83	0.67 (0.51–0.78)	0.75 (0.67–0.81)	0.72 (0.61–0.79)	0.72 (0.63–0.79)	0.91	0.73 (0.63–0.80)	0.72 (0.63–0.79)	0.73 (0.61–0.82)	0.73 (0.65–0.79)	0.97	0.70 (0.67–0.80)	0.74 (0.67–0.80)
	FGIR	0.75 (0.68–0.81)	0.56 (0.40–0.71)	0.02	0.70 (0.62–0.77)	0.48 (0.28–0.64)	0.82 (0.71–0.88)	0.76 (0.68–0.82)	0.33	0.66 (0.50–0.78)	0.68 (0.59–0.76)	0.75 (0.66–0.82)	0.76 (0.68–0.82)	0.89	0.66 (0.54–0.75)	0.67 (0.57–0.75)	0.74 (0.61–0.82)	0.77 (0.70–0.82)	0.59	0.65 (0.50–0.76)	0.69 (0.61–0.76)
	1/Fasting insulin	0.75 (0.68–0.81)	0.54 (0.35–0.69)	0.01	0.69 (0.61–0.76)	0.46 (0.26–0.62)	0.80 (0.68–0.87)	0.76 (0.68–0.82)	0.53	0.65 (0.48–0.77)	0.67 (0.57–0.75)	0.74 (0.65–0.79)	0.78 (0.68–0.82)	0.76	0.64 (0.52–0.74)	0.68 (0.58–0.76)	0.75 (0.63–0.83)	0.76 (0.69–0.81)	0.84	0.64 (0.49–0.75)	0.68 (0.59–0.75)
	WBISI	0.80 (0.74–0.84)	0.83 (0.74–0.89)	0.54	0.75 (0.68–0.81)	0.79 (0.68–0.86)	0.81 (0.70–0.88)	0.86 (0.81–0.89)	0.26	0.78 (0.66–0.86)	0.83 (0.77–0.87)	0.85 (0.78–0.89)	0.81 (0.75–0.86)	0.41	0.81 (0.74–0.86)	0.78 (0.71–0.84)	0.82 (0.73–0.61)	0.84 (0.79–0.88)	0.72	0.84 (0.76–0.89)	0.78 (0.72–0.83)

Insulin secretion	HOMA-B	0.68 (0.59–0.75)	0.61 (0.44–0.74)	0.43	0.26 (0.12–0.39	0.60 (0.43–0.73)	0.71 (0.56–0.81)	0.68 (0.58–0.75)	0.66	0.26 (0.02–0.47)	0.61 (0.50–0.70)	0.71 (0.60–0.79)	0.66 (0.55–0.74)	0.48	0.72 (0.62–0.80)	0.23 (0.07–0.38)	0.66 (0.52–0.77)	0.70 (0.61–0.76)	0.66	0.24 (0.02–0.44)	0.34 (0.20–0.46)
	IGI	0.56 (0.45–0.65)	0.65 (0.49–0.77)	0.33	−0.04 (−0.18–0.10)	0.31 (0.09–0.50)	0.55 (0.35–0.70)	0.62 (0.50–0.70)	0.53	−0.07 (−0.31–0.17)	0.22 (0.07 –0.36)	0.67 (0.55–0.76)	0.55 (0.43–0.67)	0.14	0.51 (0.36–0.63)	−0.07 (−0.23–0.10)	0.66 (0.51–0.77)	0.59 (0.48–0.68)	0.39	0.36 (0.15–0.54)	−0.04 (−0.18 –0.11)
	DI	0.49 (0.37–0.59)	0.58 (0.40–0.72)	0.36	−0.11 (−0.25–0.03)	0.13 (−0.10 –0.35)	0.49 (0.27–0.65)	0.54 (0.42–0.65)	0.61	−0.12 (−0.35–0.12)	0.04 (−0.12–0.20)	0.64 (0.51–0.73)	0.41 (0.27–0.54)	0.01	0.30 (0.13–0.46)	−0.09 (−0.25 –0.08)	0.61 (0.44–0.73)	0.48 (0.36–0.58)	0.19	0.17 (−0.05–0.38)	−0.07 (−0.21 –0.08)

## Data Availability

The data used to support the findings of this study are available from the corresponding author upon request.

## Supplementary Materials

Supplementary Materials Table S1: breakdown of demographics of patient population by sex, race, and pubertal stage.
