# Supplementary material for: The Reproducibility and Reliability of Insulin Sensitivity and Secretion Indices in Children and Adolescents
Source: Pediatr Diabetes. 2024 Apr 30;2024:2136173. doi: 10.1155/2024/2136173 (PMC11671114; doi:10.1155/2024/2136173)
Supplement: Supplementary Materials — Table S1: breakdown of demographics of patient population by sex, race, and pubertal stage. [file 2136173.f1.docx]

Supplemental Table 1. Patient Demographics

|  | **Total Patients n =257** | **Overweight/Obese n = 186** | **Normal Weight n = 71** |
| --- | --- | --- | --- |
| Sex | Male | 44% (81) | 51% (36) |
|  | Female | 56% (105) | 49% (35) |
| Race | White | 57% (106) | 68% (48) |
|  | Black | 16% (29) | 21% (15) |
|  | Other | 27% (51) | 11% (8) |
| Puberty | Pre-midpuberty | 27% (50) | 38% (27) |
|  | Post-midpuberty | 73% (136) | 62% (44) |

**Supplemental Table 1:** Breakdown of demographics of patient population by sex, race, and stage of puberty.
